# SMART: preliminary efficacy, feasibility and acceptability of a theory-informed digital intervention for metabolic health in people with schizophrenia and related disorders

**DOI:** 10.1192/bjo.2026.11032

**Published:** 2026-04-27

**Authors:** Urska Arnautovska, Gabrielle Ritchie, Nicole Korman, Anish Menon, Alyssa Milton, Marlien Varnfield, Jaimon Kelly, Pieter Jansen, Andrea Baker, Steve Kisely, Anthony Russell, Dan Siskind, Mike Trott

**Affiliations:** Faculty of Health, Medicine, and Behavioural Sciences, The University of Queensland, Brisbane, Australia; Metro South Addiction and Mental Health Services, Brisbane, Australia; Queensland Centre for Mental Health Research, Wacol, Australia; Department of Diabetes and Endocrinology, Princess Alexandra Hospital, Brisbane, Australia; Faculty of Medicine and Health, University of Sydney, Sydney, Australia; ARC Centre of Excellence for Children and Families Over the Life Course, University of Queensland, Indooroopilly, Australia; Commonwealth Scientific and Industrial Research Organisation, Brisbane, Australia; Centre for Online Health, The University of Queensland, Melbourne, Australia; School of Public Health and Preventive Medicine, Monash University, Melbourne, Australia; Centre for Online Health – Centre for Health Services Research, Faculty of Health, Medicine and Behavioural Science, The University of Queensland, Brisbane, Australia; Endocrinology and Diabetes, The Alfred Hospital, Melbourne, Australia

**Keywords:** mHealth, psychosis, mobile phone, metabolic syndrome, co-design

## Abstract

**Background:**

People with schizophrenia spectrum disorders (SSD) experience high rates of type 2 diabetes (T2D), mainly due to antipsychotic medication side-effects and lifestyle factors (e.g. suboptimal nutrition and physical inactivity). Digital technologies may reduce T2D risk by complementing face-to-face and pharmacological treatments, through the provision of flexible and personalised psychoeducation and behavioural prompts tailored to end-users.

**Aims:**

This study tested the preliminary efficacy of the Schizophrenia and diabetes Mobile-Assisted Remote Trainer (SMART), a co-designed text message-facilitated intervention, designed to reduce the risk and/or improve self-management of T2D, along with its acceptability and feasibility.

**Method:**

Using an uncontrolled pre–post design, 29 out-patients of an endocrinology mental health clinic and two community-based rehabilitation mental health facilities used SMART for 12 weeks. The primary outcome was patient activation, measured using the Patient Activation Measure. Secondary outcomes were combined objective cardiometabolic and self-reported health and mental health indicators. Pre–post changes were analysed with a linear mixed model, accounting for within-participant variation.

**Results:**

Significant improvements (*p* < 0.05) were detected in patient activation, confidence in diabetes self-management and general health management, health literacy and mental health recovery. High levels of acceptability and feasibility were confirmed, with recruitment, retention and adherence rates of 67.4, 92.9 and 93.0%, respectively.

**Conclusions:**

SMART is a world-first digital intervention aimed at improving metabolic health in individuals with SSD. This study provides evidence of its preliminary efficacy in self-management of metabolic health while confirming its high acceptability and feasibility, supporting expansion towards a sufficiently powered controlled trial to assess its clinical effectiveness.

People with schizophrenia spectrum disorders (SSD), including schizophrenia, schizoaffective disorder and brief psychotic disorder, die 15–20 years earlier than the general population. This is mostly due to cardiovascular disorder, driven by high rates of obesity and type 2 diabetes (T2D). T2D affects 18.9% of people with SSD,^1^ with 53.2% meeting the criteria for metabolic syndrome.^2^ Multiple factors contribute to this disparity, including high rates of obesity, unhealthy dietary patterns and physical inactivity, as well as challenges in accessing specialist care^3^ and the metabolic complications associated with the side-effects of antipsychotic medication.^4^ Although T2D presents a major health burden in this population, its prevention and management remain inadequately addressed.

To reduce the morbidity and mortality associated with T2D, the Lancet Commission on improving physical health in people with severe mental illness, including SSD,^4^ emphasised the need for digitally delivered diabetes prevention lifestyle programmes. More recently, the Lancet Commission team emphasised the incorporation of lived-experience perspectives, theory-based motivational approaches, relevant behaviour change techniques (BCTs) and interventions that target multiple lifestyle behaviours simultaneously.^5^ Digital technologies using a low-tech delivery format, such as short message system (SMS), that are tailored to the needs of people with SSD may be particularly well suited, particularly given the cognitive and motivational challenges associated with SSD.^6^ Text message-delivered prompts and behavioural nudges can be readily tailored to address these challenges, by providing frequent reminders and evidence-based psychoeducation to improve healthy lifestyle behaviour, illness self-management and glycaemic control.^7^ Systematic reviews demonstrate that mobile phone-based interventions are feasible and acceptable for people with SSD,^8^ with growing evidence of their potential benefits emerging from low- and middle-income countries (LMICs). Although their use to date has largely been limited to Western countries, early examples of the interventions that have been proven to change mental health outcomes are already in line to be reimbursed by health insurance companies and prescribed as digital therapeutics.^9^

In the absence of digital interventions targeting metabolic health tailored to people with SSD, we co-designed SMART (Schizophrenia and diabetes Mobile-Assisted Remote Trainer) and demonstrated its acceptability and feasibility in a 4-week, mixed-methods pilot study of the SMART prototype.^10^ SMART aims to prevent T2D and improve its self-management by providing personalised, semi-interactive psychoeducation and behavioural and motivational nudges targeting key diabetes self-care behaviours (e.g. physical activity, nutrition). These components are designed to facilitate positive health behaviour change and increase patient activation, including skills, knowledge and confidence in self-managing one’s health.

The current study aimed to evaluate the preliminary efficacy of SMART on participants’ skills, health knowledge and confidence (patient activation). A secondary aim was to explore preliminary efficacy on cardiometabolic health and mental health outcomes while confirming its usability, acceptability and feasibility in a larger sample of people with SSD who are at risk of T2D or have T2D.

## Method

### Design

This study was part of a larger, mixed-methods study that explored the preliminary efficacy, feasibility and acceptability of SMART over 12 weeks. The current study presents results of the clinical assessments with participants who used SMART for 12 weeks, in addition to their usual psychiatric treatment. Results reporting on the qualitative data based on interviews with participants are published elsewhere.^11^

### Participants and recruitment

**Fig. 1:**
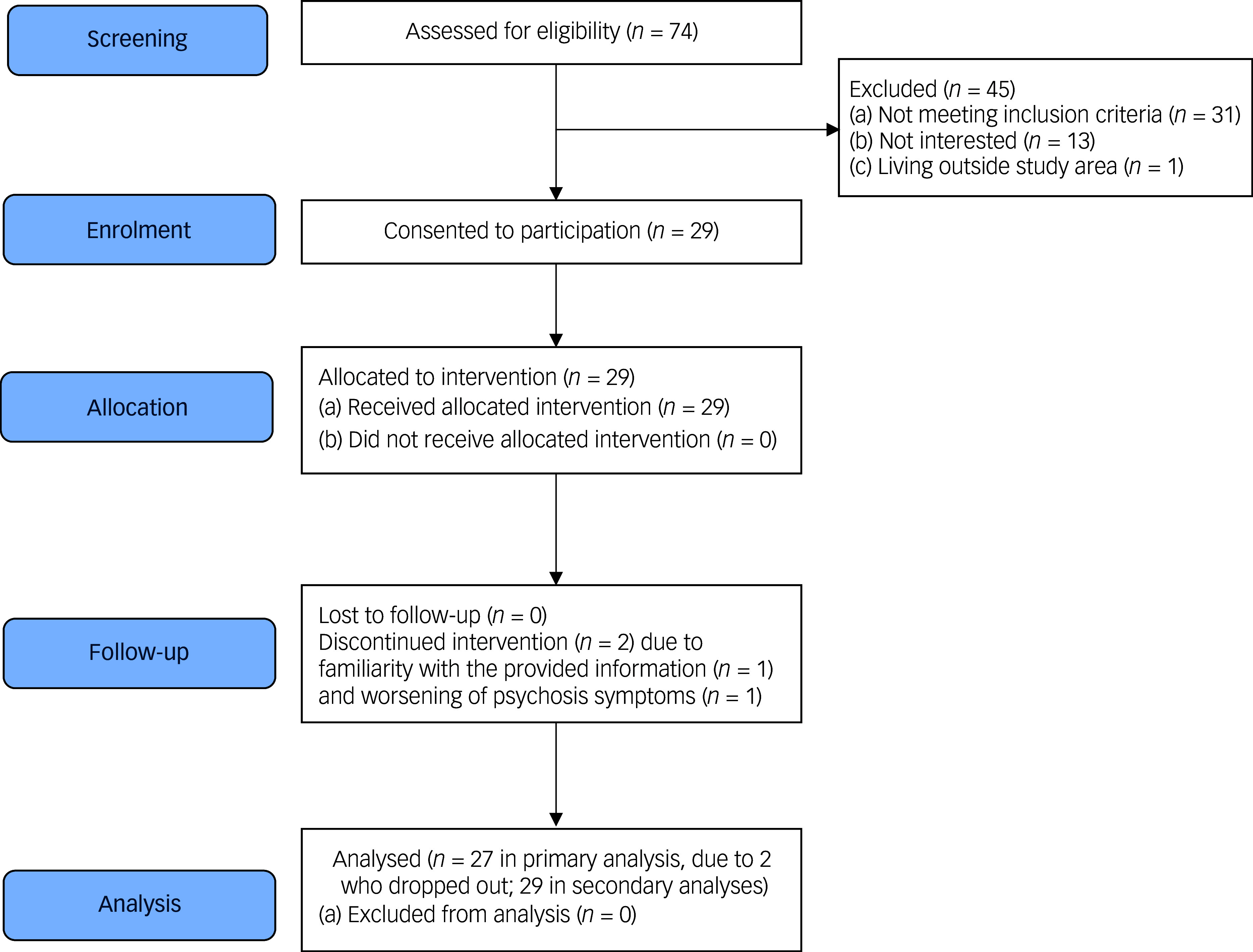
Consolidated Standards of Reporting Trials flow diagram.

Participants were recruited from three sites within the Metro South Addiction and Mental Health Services in metropolitan Brisbane, Australia, including an out-patient endocrinology clinic and two residential, community-based mental health rehabilitation facilities, between September 2024 and January 2025. Clinicians identified potential participants based on the study inclusion/exclusion criteria and referred them to research staff, who then organised a face-to-face meeting with potential participants. Research staff conducted the screening/eligibility assessment and consenting process. Verbal consent was witnessed and formally recorded. The inclusion criteria were (a) aged 18 years or older; (b) clinical diagnosis of SSD according to DSM-5, as recorded in the medical records; (c) at risk of T2D (having a diagnosis of metabolic syndrome^12^ or pre-diabetes, based on clinician-determined diagnosis documented in the clinical chart) or had T2D (based on clinical records and established by blood test); (d) able to provide informed consent; and (e) able to read/understand English. The exclusion criteria were (a) experiencing an acute relapse of psychiatric symptoms and (b) unable to use a mobile phone. Given that the current study did not require hypothesis testing, a sample size of 30 was chosen *a priori*, with the aim of informing the required sample size in a future, sufficiently powered, controlled trial. The flow of participants’ screening and recruitment is presented in a Consolidated Standards of Reporting Trials diagram (Fig. 1).

Having obtained consent to participate, the research staff set up an individual’s profile in the secure online distribution system – purposefully built and integrated within the Commonwealth Scientific and Industrial Research Organisation’s technical systems. The authors assert that all procedures contributing to this work comply with the ethical standards of the relevant national and institutional committees on human experimentation, and with the Helsinki Declaration of 1975 as revised in 2013. Ethics approval was obtained by the Metro South Human Research Ethics Committee (no. HREC/2023/QMS/100469).

### Intervention

SMART is a digital, metabolic health-focused, lifestyle intervention delivered via automated text messages that are sent to users’ mobile phones up to six times per week. It includes four core modules/topics (i.e. nutrition, weight management, physical activity and stress coping) and two optional modules (i.e. smoking/vaping cessation and blood glucose level monitoring).^10^ The wording of the messages is informed by the Self-Determination Theory^13^ and Social Cognitive Theory,^14^ with the intention to increase a person’s internal motivation and sense of agency and competence in managing their health. Additionally, each text message incorporates relevant BCTs, such as goal setting, planning and social support, from the BCT taxonomy (V1),^15^ which were selected based on previous studies assessing the efficacy of BCTs for improving lifestyle behaviours.^16,17^ The decisions guiding disorder-specific tailoring of the frequency, format and content of the text messages to accommodate the cognitive and motivational difficulties associated with SSD are detailed in a previous publication.^10^ For example, the wording in the text messages was kept to a minimum and limited to only one action per text message, to compensate for attentional deficits, whereas the initial frequency of the text messages (six messages per week) was set to assist with challenges in planning known in this population.

SMART enables personalisation to users’ preferences. In the text messages, users are addressed by their preferred name, able to select their preferred time of day to receive the message, rank the core modules in order of importance for their health and to choose one/both/neither of the optional modules. Modules ranked first and second are allocated two text messages per week, and those ranked third and fourth are allocated one text message per week (total six per week). An additional text message is sent weekly for either of the two additional modules and, if chosen, increases the number of weekly text messages to a maximum of eight. Possible replies to the text messages include Yes (indicating positive engagement in target behaviour), No or Unsure, with a response prompting an automatic reply (distinct for ‘Yes’ and ‘No/Unsure’), which may include a hyperlink to a website relevant to the message content (e.g. recipes for healthy diet). In the case of no reply, an automatic response was sent after 3 h encouraging the participant to respond. If a participant did not respond to any of the text messages for three consecutive days, an automated alarm message was sent via email to the researchers, who would then call the participant to enquire about the reasons for non-responding.

### Data collection

Baseline and 12-week endpoint assessment data were collected face-to-face at a participant’s residence by research staff, who provided the participant with a signed blood collection form for fasting bloods. Participants were asked to attend a local blood collection centre, and provided consent for the research team to review their blood results, as well as their prescribed medications, via medical records. They were also asked about their demographic and clinical information, including gender, age, level of education, living situation, smoking status, primary diagnosis and pre-diabetes/diabetes or metabolic syndrome diagnosis (confirmed with a chart review). Participants’ cognitive capacity was assessed at baseline using the Montreal Cognitive Assessment (MoCA).^18^ During baseline assessment, the research staff assisted participants in saving the phone number of the SMART system in their mobile phone. Assessments at 4 and 8 weeks were conducted by the research staff over the phone (rather than in person, to reduce the burden on both participants and research staff), to check for any technical issues experienced in the previous 4 weeks. Adverse events and serious adverse events were monitored at each assessment time point, documented, reviewed and discussed with the principal investigator (U.A.) and clinical lead (D.S.).

### Outcomes

In line with SMART’s target of increasing an individual’s autonomy and confidence in regard to effective self-management of diabetes and engagement in health behaviours critical for diabetes risk, the primary outcome was preliminary efficacy, measured using the Patient Activation Measure (PAM-13)^19^ at baseline and week 12. Secondary outcomes of clinical efficacy included physical health variables (i.e. blood pressure, waist circumference, weight, height, body mass index (BMI), blood glucose monitoring); diabetes self-management Skills, Confidence and Preparedness Index (SCPI; administered only to those with T2D);^20^ Simple Physical Activity Questionnaire (SIMPAQ);^21^ Mediterranean Diet Adherence Screener (MEDAS);^22^ Pittsburgh Sleep Quality Index (PSQI);^23^ Clinical Global Impression-severity (CGI-S);^24^ Depression Anxiety Stress Scales (DASS-21);^25^ Health Literacy Questionnaire (HLQ);^26^ mental health recovery (Recovery Assessment Scale–domain and stages, RAS-DS);^27^ stage of behaviour change; and nutrition and physical activity self-efficacy.^28^ Details of these measures across all assessment times are listed in Supplementary Tables 1 and 2. A wide range of assessments was chosen, due to the possibility that improvements in self-management could have beneficial effects on both physical and mental health.

Acceptability was assessed via user experience measured at weeks 4 and 12 by the System Usability Scale (SUS), a 10-item questionnaire used to measure the perceived usability of a digital system/product, with a 5-point response scale.^29^ Based on the average SUS score across 500 previous studies,^30^ the following grading of raw SUS scores was applied in the current study: grade A (score >80.3, above average); grade B (score 74.0–80.2, higher perceived usability than 70% of all other products tested); and grade C (score 68.0–73.9, average usability). Feasibility was assessed via (a) recruitment (proportion of eligible participants who consented to participate in the study); (b) attrition (number of participants who completed the study versus those who dropped out); (c) adherence (number (%) of Yes, No or Unsure response text messages across weeks, and number of alarm messages resulting from 3 days of not responding); and (d) retention (proportion of participants who remained in the study until the 12-week end-point). Furthermore, based on cut-offs applied in previous studies using digital interventions in people with SSD,^31,32^ adherence was classified as either ‘good’ (responding to ≥50% of all text messages over the study period) or ‘acceptable’ (a minimum of 33% response rate). An adapted, shorter version of three measures evaluating the implementation of the intervention, including Acceptability of Intervention Measure (AIM), Intervention Appropriateness Measure (IAM) and Feasibility of Intervention Measure (FIM),^33^ was also administered.

Participants were reimbursed for their time in completing assessments (AUD $50 gift voucher for both baseline and end-point), and for collection of fasting bloods (AUD $25 gift voucher for each assessment time).

### Statistical analysis plan

All statistical analyses were conducted in R (The R Foundation, Vienna, Austria; https://www.r-project.org/). Regarding pre–post outcomes, these were analysed using a linear mixed model with fixed effects for time, age and gender, and a random intercept for within-participant variation. Standard errors were derived using the Kenward–Roger approach. Missing data for mixed models were implicitly imputed under the assumption of missing at random. Complete case analyses were also conducted as a sensitivity measure. To investigate SMART feasibility, we calculated the rates of recruitment, attrition, adherence and retention over 12 weeks.

## Results

The cohort consisted of 29 participants; 41.4% were female, with a median age of 44 years. All participants had a diagnosis of either schizophrenia or schizoaffective disorder. Regarding their metabolic health, 58.6% had a diagnosis of T2D, 6.9% had pre-diabetes and 58.6% met the criteria for metabolic syndrome.^12^ The mean BMI was 37 kg/m^2^ (interquartile range (IQR) 30.7–41.8), corresponding to obesity. Based on total MoCA score, 48.3% were classified within the normal range of cognition, with 31.0 and 20.7% demonstrating mild or moderate cognitive impairment, respectively. Full descriptive information can be found in Table 1.

**Table 1:** Descriptive characteristics at baseline (*n* = 29)

Variables	*n* (%)
Demographic characteristics	
Gender	
Male	17 (58.6)
Female	12 (41.4)
Age, years [median, IQR]	44 [36–52]
Living situation	
Supported housing	8 (27.6)
Independent (living alone)	12 (41.4)
Independent (living with others)	9 (31.0)
Education	
Completed Year 9 or below	3 (10.3)
Completed Year 10	4 (13.8)
Completed Year 11	2 (6.9)
Completed Year 12	8 (27.6)
Higher education degree	12 (41.4)
Clinical characteristics	
Metabolic health	
T2D	17 (58.6)
Pre-diabetes	2 (6.9)
Metabolic syndrome	17 (58.6)
GLP-1RA prescription	5 (17.2)
Smoking status	
Never	11 (37.9)
Former	5 (17.2)
Current	13 (44.8)
Cigarettes per day	15 (0–20)
Anthropometrics	Median [IQR]
Weight, kg	104.6 [96.3–124.9]
BMI	37 [30.7–41.8]
Waist circumference, cm	121 [109–125]
Hip:waist ratio	1.0 [1.0–1.0]
Cardiometabolic markers	
Blood pressure	
Systolic	116 [109–127]
Diastolic	78 [71–80]
HbA1c	5.9 [5.5–6.7]
Fasting glucose	5.9 [5.2–7.0]
Fasting triglycerides	2.0 [1.5–2.6]
Fasting HDL	1.07 [0.9–1.3]
Fasting LDL	2.15 [1.5–2.8]
Total cholesterol	4 [3.3–4.7]
Self-management and health literacy	
Diabetes self-management (SCPI)^a^	
SCPI skills	4.3 [3.3–5.3]
SCPI confidence	4.8 [4.2–5.4]
SCPI preparedness	5.3 [5.0–5.5]
SCPI total	4.8 [4.2–5.2]
Health literacy (HLQ)	
Domain 3 total	2.8 [2.6–3.0]
Nutrition self-efficacy	15.0 [13.0–16.0]
Physical activity self-efficacy	13.0 [10.0–15.0]
Patient activation (PAM)	
PAM-13 total	39.0 [37.0–41.0]
PAM-13 total calibrated	53.2 [51.0–60.6]
Mental health recovery (RAS-DS)	
Domain 1 total	17.0 [16.0–19.0]
Domain 3 total	21.0 [17.0–24.0]
Lifestyle behaviours	
Sedentary behaviours, h/day	10.0 [7.0–12.0]
MVPA, min/day	0.5 [0.4–1.0]
MEDAS	5 [3–7]
Psychological characteristics	
Sleep	
PSQI sleep quality	1.0 [1.0–1.0]
PSQI day dysfunction	1.0 [1.0–1.0]
Mental health	
DASS depression	6.0 [4.0–14.0]
DASS anxiety	6.0 [2.0–12.0]
DASS stress	8.0 [4.0–16.0]
Cognition	
Total MoCA score	25 [21–27]
MoCA categories	
Normal cognition (%)	14 (48.3)
Mild cognitive impairment (%)	9 (31.0)
Moderate cognitive impairment (%)	6 (20.7)
Severe cognitive impairment (%)	0 (0.0)

Data shown as either median [interquartile range] or *n* (percentage).

T2D, type 2 diabetes; GLP-1RA, glucagon-like peptide-1 receptor agonist; IQR, interquartile range; BMI, body mass index; HbA1c, glycated haemoglobin; HDL, high-density lipoprotein; LDL, low-density lipoprotein; SCPI, Skills, Confidence and Preparedness Index; HLQ, Health Literacy Questionnaire; PAM, Patient Activation Measure; RAS-DS, Recovery Assessment Scale – domain and stages; MVPA, moderate to vigorous physical activity; MEDAS, Mediterranean Diet Adherence Screener; PSQI, Pittsburgh Sleep Quality Index; DASS, Depression Anxiety Stress Scales; MoCA, Montreal Cognitive Assessment.

a. Administered in those with T2D.

There were two participants who withdrew during the study: one in week 4, due to perceived redundancy of the content, and one in week 11, owing to reportedly increased paranoia and anxiety related to anticipation of the end-point assessment, which was recorded as an adverse event.

Weight management was most frequently ranked as the highest-priority module (*n* = 11), followed by physical activity (*n* = 11). Nutrition was ranked among the top three highest-priority modules by eight participants, whereas coping with stress most often ranked as third (*n* = 9) or fourth (*n* = 8). Monitoring blood glucose levels was selected by 12 participants, and 5 of the 13 active smokers chose the quitting smoking/vaping module.

### Preliminary efficacy of SMART

Regarding pre–post intervention changes, total PAM scores significantly increased, from 39.0 pre-intervention to 41.9 post-intervention (*p* = 0.005), with significant increases also observed in PAM calibrated scores (56.7 to 63.5, *p* = 0.014). RAS-DS domain 1 (‘Doing things I value’) scores also significantly increased, from 18.0 to 19.1 (*p* = 0.022), along with SCPI total (4.6–5.2, *p* = 0.017), SCPI confidence (4.6–5.3, *p* ≤ 0.001) and HLQ health literacy domain 3 ‘Actively managing my health’ (2.8–3.0, *p* = 0.032). Notably, effect sizes of changes in health literacy (HLQ) and mental health recovery (RAS-DS) were medium to large (*d* = 0.49 and 0.45, respectively), whereas those in nutrition and physical activity self-efficacy scores were small to medium (*d* = 0.28 and 0.40, respectively). All other pre–post scores, including anthropometrics, objective cardiometabolic markers (obtained via blood tests), lifestyle behaviours and psychiatric symptoms, were non-significant (see Table 2 and Fig. 3). Out of five participants who chose the optional module on quitting smoking/vaping, one quit smoking during the study duration.

**Fig. 2:**
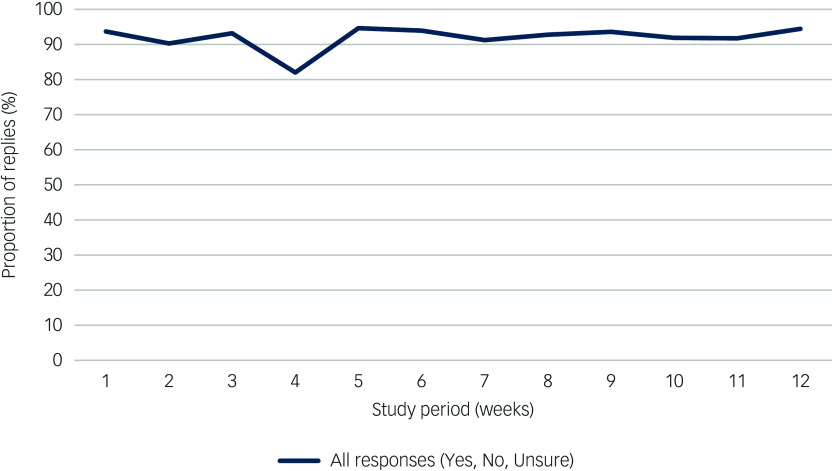
Types of response to the Schizophrenia and diabetes Mobile-Assisted Remote Trainer (SMART) text message across the 12-week period (*n* = 27).

**Fig. 3:**
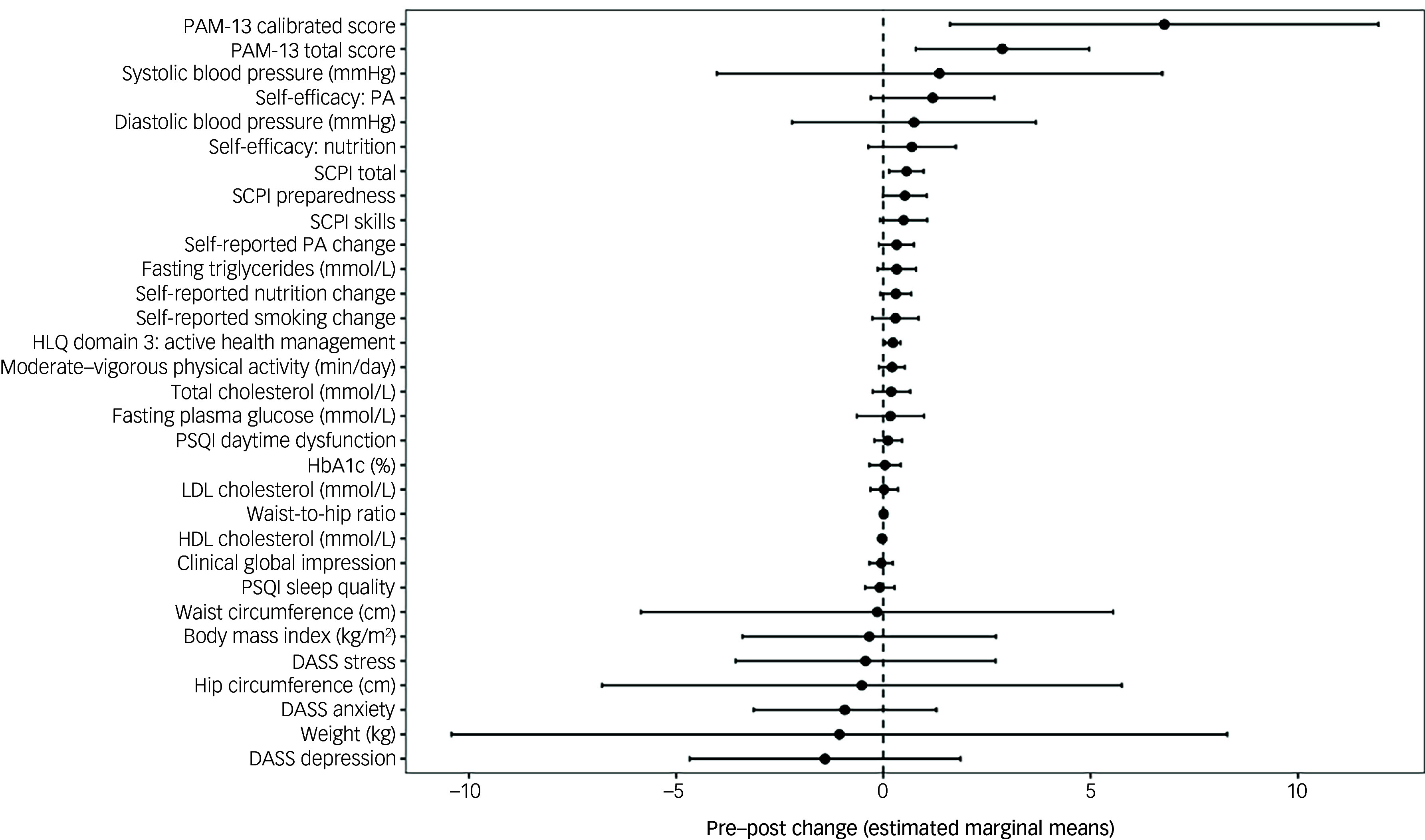
Effect sizes of changes in outcomes following 12 weeks of using the Schizophrenia and diabetes Mobile-Assisted Remote Trainer (SMART). PAM-13, Patient Activation Measure; SCPI, diabetes self-management ‘Skills, Confidence and Preparedness Index’; PA, physical activity; HLQ, Helath Literacy Questionairre; PSQI, Pittsburgh Sleep Quality Index; HbA1c, Glycated haemoglobin; LDL, low-density lipoprotein; HDL, high-density lipoprotein; DASS, Depression Anxiety Stress Scales.

**Table 2:** Comparison of pre and post scores following 12 weeks of using the Schizophrenia and diabetes Mobile-Assisted Remote Trainer (SMART) intervention

Outcomes		Pre (95% CI)	Post (95% CI)	Difference (95% CI)	*p*-value	Cohen’s *d*
Self-management and health literacy						
Patient activation		PAM-13 total*	38.98 (36.88–41.08)	41.85 (39.71–44)	2.87 (0.95–4.80)	0.005
		PAM-13 total calibrated*	56.67 (51.5–61.84)	63.45 (58.13–68.76)	6.78 (1.48–12.08)	0.014
Diabetes self-management		SCPI skills	4.07 (3.5–4.64)	4.56 (4.01–5.11)	0.49 (−0.19 to 1.17)	0.144
		SCPI confidence*	4.61 (4.22–5.01)	5.28 (4.89–5.66)	0.67 (0.32–1.01)	<0.001
		SCPI preparedness	5.12 (4.59–5.65)	5.64 (5.12–6.16)	0.52 (−0.06 to 1.10)	0.077
		SCPI total*	4.6 (4.19–5.02)	5.16 (4.75–5.56)	0.56 (0.11–1.00)	0.017
Health literacy HLQ domain 3*		2.81 (2.63–2.99)	3.04 (2.86–3.23)	0.23 (0.02–0.44)	0.032	0.49
Nutrition self-efficacy total		14.46 (13.41–15.52)	15.16 (14.07–16.24)	0.69 (−0.34 to 1.72)	0.179	0.28
Physical activity self-efficacy total		12.86 (11.37–14.35)	14.05 (12.53–15.57)	1.19 (−0.08 to 2.47)	0.066	0.40
Lifestyle behaviours						
Sedentary behaviours (min/day)		9.89 (8.68–11.09)	10.95 (9.7–12.2)	1.07 (−0.45 to 2.58)	0.160	0.3
MVPA (min/day)		0.71 (0.4–1.03)	0.92 (0.6–1.24)	0.21 (−0.05 to 0.47)	0.114	0.32
MEDAS		4.54 (3.76-5.33)	5.15 (4.35-5.95)	0.61 (−0.15 to 1.36)	0.111	0.35
Psychological characteristics						
Symptom severity						
CGI-S		3.73 (3.44–4.01)	3.67 (3.38–3.97)	−0.05 (−0.37 to 0.27)	0.727	0
Sleep						
PSQI sleep quality		1.08 (0.72–1.43)	0.99 (0.63–1.35)	−0.09 (−0.41 to 0.24)	0.587	−0.09
PSQI day dysfunction		1.13 (0.79–1.47)	1.24 (0.9–1.59)	0.11 (−0.25 to 0.47)	0.522	0.16
Mental health						
DASS depression		8.80 (5.53–12.07)	7.39 (4.09–10.69)	−1.41 (−3.37 to 0.55)	0.151	−0.28
DASS anxiety		7.95 (5.75–10.16)	7.03 (4.78–9.27)	−0.93 (−2.67 to 0.82)	0.286	−0.18
DASS stress		9.18 (6.04–12.32)	8.75 (5.57–11.93)	−0.43 (−2.53 to 1.67)	0.679	−0.08
Mental health recovery (RAS-DS)						
Domain 1 total*		17.97 (16.83–19.11)	19.14 (17.98–20.3)	1.18 (0.18–2.17)	0.022	0.45
Domain 3 total		20.43 (18.74–22.13)	20.53 (18.8–22.26)	0.10 (−1.43 to 1.62)	0.898	0
Anthropometrics						
Weight (kg)		107.48 (98.12–116.84)	106.42 (97.06–115.79)	−1.06 (−2.18 to 0.07)	0.064	−0.37
BMI		36.7 (33.65–39.76)	36.36 (33.3–39.42)	−0.34 (−0.73 to 0.05)	0.083	−0.34
Waist circumference (cm)		117.41 (111.71–123.11)	117.26 (111.51–123.01)	−0.15 (−3.28 to 2.98)	0.922	−0.01
Hip:waist ratio		0.97 (0.96–0.99)	0.98 (0.96–1)	0.01 (−0.02 to 0.03)	0.612	0.05
Cardiometabolic markers						
Blood pressure						
Systolic		115.78 (110.4–121.16)	117.13 (111.64–122.63)	1.35 (−3.43 to 6.14)	0.566	0.14
Diastolic		77.75 (74.82–80.69)	78.49 (75.49–81.49)	0.74 (−1.76 to 3.24)	0.550	0.12
HbA1c		6.1 (5.71–6.48)	6.13 (5.75–6.52)	0.04 (−0.21 to 0.28)	0.746	0.08
Fasting glucose		6.37 (5.55–7.18)	6.54 (5.72–7.36)	0.17 (−0.43 to 0.78)	0.563	0.15
Fasting triglycerides		2.06 (1.6–2.53)	2.39 (1.91–2.86)	0.32 (−0.02 to 0.67)	0.064	0.37
Fasting HDL		1.08 (0.99–1.18)	1.05 (0.96–1.15)	−0.03 (−0.07 to 0.01)	0.105	−0.34
Fasting LDL		2.06 (1.73–2.39)	2.08 (1.74–2.41)	0.02 (−0.20 to 0.24)	0.865	0.03
Total cholesterol		4.04 (3.59–4.5)	4.24 (3.77–4.7)	0.19 (−0.14 to 0.53)	0.250	0.23

PAM, Patient Activation Measure; SCPI, Skills, Confidence and Preparedness Index; HLQ, Health Literacy Questionnaire; MVPA, moderate to vigorous physical activity; MEDAS, Mediterranean Diet Adherence Screener; CGI-S, Clinical Global Impression-Severity; PSQI, Pittsburgh Sleep Quality Index; DASS, Depression Anxiety Stress Scales; RAS-DS, Recovery Assessment Scale – domain and stages; BMI, body mass index; HbA1c, glycated haemoglobin; HDL, high-density lipoprotein; LDL, low-density lipoprotein. *, type 2 diabetes significant pre–post difference *p ≤* 0.05.

### Acceptability of SMART

Due to the non-normality of the acceptability outcome, measured via SUS (Shapiro–Wilk = 0.003), data are presented as median and IQR. The median SUS score was 75 (IQR 72.5–80.0), ranging from 62.5 to 102.5, indicating grade B usability (i.e. higher perceived usability than 70% of all other tested products in 500 previous studies tested^30^). High acceptability of SMART was also supported by participants’ scores on the 5-point AIM (median 5, range 4–5).

### Feasibility of SMART

The recruitment rate was 67.4% (29/43), with low attrition (2 out of 29, 6.9%; see Fig. 1). Reasons for non-participation among 13 eligible referrals from the endocrinology clinic (i.e. those who met the study criteria) were unrelated to intervention delivery, and primarily reflected competing life demands (‘too much on my plate’). SMART was rated as being feasible and appropriate, with median scores of 4 (range 3–5) on both FIM and IAM. Adherence was high, with a mean text message response rate (i.e. proportion of responses Yes, No or Unsure to text message) of 93% during the 12-week period (Fig. 2), and nine instances of 3-day non-response across six participants. Engagement was stable (≥90%) across all 12 weeks, with a slight decrease in week 4 (82%) attributable to either the Christmas period or running out of phone credit. The proportion of ‘Yes’ replies (indicating positive engagement in target behaviour) decreased modestly, from 71% in week 1 to 58% in week 12 (Supplementary Table 3). Across all modules, response rates ranged between 90% (physical activity) and 98% (monitoring blood glucose levels), exceeding the 50% pre-defined adherence threshold for all modules (Supplementary Table 4).

## Discussion

This study investigated the preliminary efficacy, along with acceptability and feasibility, of a co-designed digital intervention aimed at improving metabolic health in people living with SSD who are either at risk of developing, or have already developed, T2D. Whereas digital mental health interventions are considered a promising add-on treatment for various mental health conditions, including schizophrenia, there are currently no digital interventions tailored to the needs of this population that target physical health. As such, SMART has the potential to fill a critical gap in addressing the significant physical health inequality experienced by people with SSD – specifically, their high rates of T2D, one of the key drivers of cardiometabolic-associated morbidity and mortality.^4^

Our study results provide preliminary evidence of the efficacy of SMART; this was demonstrated by statistically significant improvements in five outcomes, including primary outcome and patient activation. This is a critical finding given that prior evidence has shown that health outcomes can be improved by increasing levels of patient activation, an established predictor of lifestyle behaviours crucial to metabolic health (e.g. physical activity), metabolic indicators (e.g. cholesterol and progression from pre-diabetes to T2D), T2D self-management and health service use in the general population and those with SSD.^34^ Furthermore, given that individuals with SSD often struggle to communicate their needs and take an active role in managing their metabolic health,^35^ slowing the development and progression of T2D through supporting one’s skills, knowledge and confidence relevant to metabolic health has significant clinical and resource implications. This evidence supports a mechanistic pathway of change targeted by SMART that may impact lifestyle behaviour and change in metabolic health indicators through increasing a person’s level of patient activation. This also highlights its potential for contributing to positive effects on a broad range of physical and mental health indicators, including healthcare service use, that are known to be impacted by patient activation.^34^

Statistically significant improvements were also found in health literacy and mental health recovery, both of medium to large effect sizes. Specifically, among those diagnosed with T2D, confidence in diabetes self-management and overall diabetes self-management also increased significantly. The breadth of these changes is probably reflective of the diversity of content included in SMART. The modules ranged from nutrition, physical activity and weight management to stress-coping (in addition to optional monitoring of blood glucose levels and smoking/vaping cessation modules). In addition, a range of BCTs were embedded within the text messages, including social support, information about health consequences, goal-setting and problem-solving.^10^ This corroborates previous evidence showing greater efficacy of lifestyle interventions in people with severe mental illness, including schizophrenia, when these included the BCTs of goal-setting and planning.^36^

Whereas SMART also aimed to improve users’ engagement in healthy lifestyle behaviours critical for the development and self-management of T2D, participants reported no significant changes in their levels of physical activity engagement, or nutrition, following 12 weeks of SMART usage; however, one of the five participants who chose the quitting smoking/vaping module quit smoking. The lack of changes in lifestyle behaviours is reflective of our pilot study results.^10^ Contrary to our expectation that participants would report increased engagement in these behaviours over time, the proportion of ‘Yes’ replies gradually decreased over the 12 weeks for 13%. This may be due to either participants providing more socially desirable responses at the start of the study, more objective responding to the text message-facilitated prompts as their knowledge about target behaviour improved or a decreasing number of text messages across the study period, affecting the proportion of each response option. This finding highlights the importance of using objective measurements of lifestyle behaviours, specifically of physical activity and sedentary behaviour (e.g. via accelerometry),^37^ to overcome the limitations of self-report, which is well known in schizophrenia samples, and considering even greater personalisation of the content of the text messages, possibly using Artificial Intelligence features, may also maximise the intervention’s impact. Furthermore, given the absence of significant changes in objective cardiometabolic markers, extending access to SMART (e.g. to 24 weeks) could support more sustained engagement in key lifestyle behaviours. Follow-up assessments (e.g. 12 weeks post-intervention) may also help in the detection of the potential effects of SMART on cardiometabolic outcomes that are slower to change.

We also found that SMART showed high levels of acceptability and feasibility for participants, which confirms the findings from our pilot testing.^10^ Acceptability, as measured with SUS, indicated that SMART was perceived better – in terms of its ease of usability, complexity and prior learning required to use it – than 70% of products in previously tested studies.^30^ High acceptability was also reflected in high mean AIM scores, and is supported by strong evidence of its feasibility. Furthermore, the adherence rate (proportion of replies to SMART text messages) indicates excellent engagement of participants, which was sustained across 12 weeks except for the Christmas period. Reassuringly, these adherence rates were consistent with the level of engagement observed in our previous 4-week pilot study.^10^

Compared with 26 other existing digital interventions trialled among people with SSD,^8^ where pooled rates of recruitment and retention were 57.4 and 87.6%, respectively, SMART demonstrated superior acceptability and feasibility over the 12-week study period, with only a fifth of participants having 9 instances of temporarily not responding to text messages. Potential reasons for such favourable feasibility metrics include its low-tech delivery format, requiring no prior learning about the use of SMS technology (the same could not be said for the use of smartphone apps); the absence of a requirement for internet data to receive or read text messages (although participants had the option to access further information via web-links included in 23.6% of response messages across four core modules); and low cognitive demand, ensured by a reading age of the messages equivalent to 11–12 years.^10^ Additionally, the engagement may have been, in part, sustained through research staff-facilitated check-in phone calls (at weeks 4 and 8, and also at each 3-day non-response instance), providing participants with an opportunity to discuss any issues in regard to receiving or sending text messages and, therefore, supporting their capacity to remain engaged with SMART. The importance of incorporating human support within digital mental health interventions designed specifically for people with SSD has been noted previously.^38^ Finally, supporting the findings by Vancampfort and colleagues,^39^ who found that drop-out from physical activity interventions among people with severe mental illness was lower when interventions used autonomous (self-determined/intrinsic) motivational strategies, high adherence and retention in SMART may also have been sustained by strong grounding in self-determination concepts. Our findings provide additional evidence to inform global discussions around optimal delivery and implementation of innovative, digitally delivered treatment modalities for people living with various mental health conditions.^40^

Taken together, the positive changes demonstrated in the current study corroborate increasing evidence that external prompts and behavioural nudges, through apps and text messages, can provide reminders and practical strategies to improve T2D illness self-management among people with SSD.^7^ Considering that cognitive challenges associated with SSD can impact glucose monitoring, remembering medications and making healthy lifestyle choices – thereby undermining effective glycaemic control – SMART highlights the potential usefulness of technological support, through a low-tech SMS format, to improve lifestyle behaviours and glycaemic control. Furthermore, given the low-cost delivery of SMART, such an intervention may be well suited for implementation in LMICs, or in areas with limited internet access such as rural and remote areas where resources are typically scarce and internet coverage less reliable. Such expansion would also address significant knowledge gaps in understanding the feasibility, efficacy and long-term clinical benefits of digital health interventions in LMICs.^41^

### Strengths and limitations

To our knowledge, SMART is the first digitally delivered intervention designed to improve metabolic health tailored to people living with SSD. As such, SMART text messages are unrestricted in terms of delivery location and much easier to be scalable beyond clinic spaces compared with traditional, in-person approaches. A notable strength of SMART is its comprehensive multi-stage co-design process,^10^ ensuring that its content and delivery format reflect the needs and priorities of end-users. Furthermore, SMART text messages are theory-driven, encapsulating the principles of the Self-Determination Theory and Social Cognitive Theory, with evidence-based BCTs embedded within each message. This facilitates positive health behaviour change through increased levels of patient activation, leading to greater autonomy and internal motivation in managing one’s health. However, the validity of the hypothesised mechanisms (as informed by the premises of Self-Determination Theory and Social Cognitive Theory) would need to be tested using structural or mediation analysis in a larger sample of participants.

In the current study, we also did not assess the impact of demographic, socioeconomic or clinical characteristics of participants, who were all out-patients, on their acceptance of SMART. Given that acceptability may differ across illness stages (e.g. first-episode psychosis, treatment-resistant schizophrenia), healthcare service context (e.g. in-patient, community rehabilitation) and geographic region (e.g. metropolitan, regional/remote), it would be relevant to explore these aspects in future testing, as well as to adapt it for non-English speakers and those not proficient in English. Furthermore, whereas some response messages included links to relevant online resources (e.g. healthy recipes on the Diabetes Australia website), we were unable to measure engagement with these internet links because these websites were all hosted by external platforms. Additionally, the use of a single-arm, uncontrolled study design with small sample size precludes conclusions about causality and potential subgroup differences in the value of SMART for those with T2D against those at risk of T2D. This highlights the need for future testing of SMART in a sufficiently powered randomised controlled trial that would compare SMART with standard care or other control conditions. Extending follow-up to assess sustained behaviour change and adherence would also be beneficial.

Our findings support the preliminary efficacy of SMART across a range of outcomes – most notably in enhancing patient activation, the proposed mechanism driving lifestyle change and metabolic outcomes – justifying progression to a fully powered randomised controlled trial. Grounded in robust theoretical frameworks that foster intrinsic motivation and offer practical, evidence-based strategies for sustained lifestyle change, SMART is tailored to the needs and preferences of people with complex mental health conditions. Combined with high levels of acceptability and feasibility, this study provides compelling evidence supporting SMART’s potential for improving metabolic health in individuals with SSD, particularly in the prevention and self-management of T2D, and thus a promising opportunity to reduce the substantial cardiometabolic burden among individuals with severe mental illness. Future exploratory research should also focus on optimising the implementation of SMART across diverse healthcare settings and geographic regions, using minimal researcher-led, face-to-face assessments to better reflect real-life service delivery and support clinical effectiveness, sustainability and acceptability in routine care.

## Supporting information

10.1192/bjo.2026.11032.sm001 Arnautovska et al. supplementary material Arnautovska et al. supplementary material

## Data Availability

The data that support the findings of this study are available from the corresponding author, U.A., upon reasonable request, as per the study protocol.

## Supplementary material

The supplementary material is available online at https://doi.org/10.1192/bjo.2026.11032
