# Supplementary material for: SMART: preliminary efficacy, feasibility and acceptability of a theory-informed digital intervention for metabolic health in people with schizophrenia and related disorders
Source: BJPsych Open. 2026 Apr 27;12(3):e122. doi: 10.1192/bjo.2026.11032 (PMC13122338; doi:10.1192/bjo.2026.11032)
Supplement: Arnautovska et al. supplementary material [file S2056472426110321sup001.docx]

**Supplementary Table 1.** Description of outcome measures used in the SMART study

| Outcome measure | Brief description | | Outcomes used in the analysis | |
| --- | --- | --- | --- | --- |
| Objective measures |  | |  | |
| Physical health | Physical examination (blood pressure, waist circumference, weight, height, and BMI); BGL monitoring (if relevant). | | Mean Body Mass Index (BMI) calculated based on a person’s weight and height at baseline.  Mean metabolic parameters: body weight; blood pressure; and waist circumference.  Frequency of BGL monitoring (if relevant). | |
| Metabolic investigations | Fasting metabolic bloods - HbA1c, plasma glucose, triglycerides, HDL, LDL, and total cholesterol. | | Mean metabolic parameters on fasting bloods. | |
| Primary outcome | | | | |
| Patient activation | | Patient Activation Measure (PAM-13),^1, 2^ a 13-item scale that evaluates a patient’s ability to self-manage their chronic illness through confidence, knowledge, and skills. | | Mean PAM-13 total calibrated score.  Frequency PAM-13 level based on calibrated total score. |
| Self-management skills and lifestyle behaviour | | | | |
| Diabetes self-management | Skills, Confidence and Preparedness Index (SCPI)^3^ consists of 23 items that measure an individual’s skills, confidence, and preparedness in managing glycaemic control. | | Mean SCPI total score, mean Skills, Confidence, and Preparedness subscale scores. | |
| Physical activity | The 5-item Simple Physical Activity Questionnaire (SIMPAQ)^4^ estimates time in bed, structured exercise participation, and incidental or non-structured physical activity. | | Mean Sedentary time (min) per day (box 2)  Mean Total moderate-vigorous physical activity (MVPA) (min) per week. | |
| Diet quality | The 14-item Mediterranean Diet Adherence Screener (MEDAS)^5, 6^ measures adherence with the Mediterranean dietary pattern as one of the most validated dietary approaches that can help control blood pressure and blood sugar. | | Mean total score. | |
| Smoking and vaping status | Self-report of current and former history of smoking cigarettes/e-cigarettes. | | Proportion of sample currently smoke and previously smoked. Mean no. of cigarettes/e-cigarettes smoked per day.  Mean duration (yrs.) smoking. | |
| Sleep | Two subscales from the Pittsburgh Sleep Quality Index (PSQI),^7^ a self-reported measure of overall sleep quality assessed over a 1-month period. | | Mean Perceived Sleep Quality Total Score and Daytime Dysfunction Total Score. | |
| Nutrition self-efficacy | A 5-item measure examining social-cognitive determinants of health behaviour (Nutrition) on a 4-point scale (Strongly disagree to Strongly agree)^8^. | | Mean nutrition self-efficacy total score. | |
| Physical activity self-efficacy | A 5-item measure examining social-cognitive determinants of health behaviour (Physical activity) on a 4-point scale (Strongly disagree to Strongly agree)^8^. | | Mean physical activity self-efficacy total score. | |
| General self-efficacy | Domain 1 (Doing things I value) and Domain 3 (Mastering my illness) from the Recovery Assessment Scale – Domain and Stages (RAS‐DS)^9, 10^ which measures self-reported mental health recovery on a 4-point scale (Untrue to Completely true). | | Mean total scores for Domain 1 Domain 3. | |
| Stage of behaviour change | Questions based on the transtheoretical mode of stages of change (pre-contemplation, contemplation, preparation, action, and maintenance) for physical activity, nutrition, and smoking/vaping cessation. The target behaviours are aligned with national guidelines on physical activity and nutrition^11^. | | Number (%) of participants at each stage of behaviour change for physical activity, nutrition, and smoking/vaping cessation. | |
| Other assessments |  | |  | |
| Symptom severity | Clinical global impression-severity (CGI-S)^12^ is a clinician-rated scale which assesses the severity of mental health symptoms using a 7-point scale (‘Normal, not at all’ to ‘Among the most extremely ill patients’), reflecting the clinician’s assessment of the disease impact on the person’s global functioning. | | Mean CGI-S score. | |
| Health literacy | Domain 3 (Actively managing my health) from the Health Literacy Questionnaire (HLQ), includes five statements on a 4-point scale (Strongly agree to Strongly Disagree)^13^. | | Mean domain 3 total score. | |
| Negative emotional symptoms | Depression Anxiety Stress Scales (DASS-21)^14, 15^, 21-item measure of individual’s experience of depression, anxiety, and stress over the past week on a 4-point severity/frequency scale. | | Mean Depression, Anxiety, and Stress total scores. | |
| Acceptability and feasibility | | |  | |
| Feasibility | Proportion of eligible participants who consent to (recruitment), and complete, the 12-week study (attrition). | | Total recruitment and attrition, calculated as proportion of eligible participants who: consent to study; remain until 12-week endpoint; complete 12-week endpoint data. | |
| Acceptability | Semi-structured interviews exploring participant experiences of intervention; specifically, perceived usefulness of text messages and suggestions for improvement. Digitally recorded and transcribed verbatim. | | Thematic analysis and generation of themes based on qualitative data. | |
| Cognition |  | |  | |
| Cognitive impairment | The Montreal Cognitive Assessment (MoCA) ^16, 17^ is used for assessment of mild  cognitive impairment and includes assessments of visuospatial and executive function. | | Number (%) of participants with normal cognition, and mild, moderate, and severe cognitive impairment. | |

**Supplementary Table 2.**

| **Visit** | 0 | 1 | 2 | 3 | 4 |
| --- | --- | --- | --- | --- | --- |
|  | **Screening** | **Baseline** |  |  |  |
| **WEEK** | 0 | 0 | 4 | 8 | 12 |
| **SCREENING AND CONSENT** |  |  |  |  |  |
| Assessment of current medication incl. GLP-1 |  | X | X | X | X |
| Informed consent | X |  |  |  |  |
| Ongoing capacity | X | X | X | X | X |
| Inclusion / Exclusion criteria | X |  |  |  |  |
| **Primary outcome** |  |  |  |  |  |
| Patient activation (PAM-13) |  | X |  |  | X |
| **Secondary outcomes** |  |  |  |  |  |
| User experience (SUS) |  |  | X |  | X |
| Implementation^a^ |  |  |  |  | X |
| **Objective measures:** |  |  |  |  |  |
| HbA1c^b^ |  | X |  |  | X |
| Frequency of BGL monitoring^c^ (self-report) |  | X | X | X | X |
| Fasting metabolic blood tests |  | X |  |  | X |
| Physical observations (weight, BMI, blood pressure, waist circumference) |  | X |  |  | X |
| Height |  | X |  |  |  |
| **Self-report measures:** |  |  |  |  |  |
| Diabetes self-management (SCPI)^d^ |  | X | X | X | X |
| Health literacy (HLQ)^e^ |  | X |  |  | X |
| Physical activity self-efficacy (5 items) |  | X |  |  | X |
| Nutrition self-efficacy (5 items) |  | X |  |  | X |
| Symptom severity (CGI-S) |  | X |  |  | X |
| Diet quality (MEDAS) |  | X |  |  | X |
| Physical activity (SIMPAQ) |  | X |  |  | X |
| Smoking status (self-report) |  | X |  |  | X |
| Sleep (PSQI)^f^ |  | X |  |  | X |
| Depression & anxiety symptoms (DASS-21) |  | X |  |  | X |
| Recovery (RAS-DS)^g^ |  | X |  |  | X |
| **OTHER** |  |  |  |  |  |
| Cognition (MoCA) |  | X |  |  |  |

^a^

^a^

BMI: Body Mass Index; CGI-S: Clinical Global Impression-severity; DASS-21: Depression, Anxiety and Stress Scales; MEDAS: Mediterranean Diet Adherence Screener; HLQ: Health Literacy Questionnaire; PAM-13: Patient Activation Measure; SCPI: diabetes self-management Skills, Confidence and Preparedness Index; SIMPAQ: Simple Physical Activity Questionnaire; SUS: system Usability Scale;

^a^Implementation was measured with an adapted, short version of three measures evaluating the implementation

of the intervention, including Acceptability of Intervention Measure (AIM), Intervention Appropriateness Measure (IAM), and Feasibility of Intervention Measure (FIM).^18^

^b^ If a participant had HbA1c or PANSS measured within the previous four weeks prior to baseline assessment, we used existing results and not repeat these tests at baseline.

^c^ Relevant to participants who are required to monitor BGL.

^d^ SCPI was conducted among participants with T2DM.

^e^ Health literacy was measured using HLQ Domain 3 - Actively managing my health.

^f^ Sleep was measured using PSQI subscales Perceived Sleep Quality and Daily Disturbances.

^g^ Self-efficacy was measured using RAS-DS Domain 1 – Doing things I value, and Domain 3 – Mastering my illness.

**Supplementary Table 3.** Response rates for SMART text message across the 12-week period (*n* = 27)

| Participants’ reply | Week 1 | Week 2 | Week 3 | Week 4 | Week 5 | Week 6 | Week 7 | Week 8 | Week 9 | Week 10 | Week 11 | Week 12 | | All weeks |
| --- | --- | --- | --- | --- | --- | --- | --- | --- | --- | --- | --- | --- | --- | --- |
|  | N (%) | N (%) | N (%) | N (%) | N (%) | N (%) | N (%) | N (%) | N (%) | N (%) | N (%) | N (%) | N (%) | |
| Yes | 124 (71) | 98 (56) | 87 (49) | 104 (52) | 88 (59) | 81 (55) | 76 (55) | 79 (57) | 63 (59) | 65 (59) | 77 (71) | 74 (69) | 1,016 (58) | |
| No | 25 (14) | 40 (23) | 63 (36) | 45 (22) | 41 (28) | 44 (30) | 38 (28) | 34 (25) | 32 (29) | 29 (26) | 15 (14) | 17 (16) | 423 (24) | |
| Unsure | 14 (9) | 20 (11) | 14 (8) | 15 (8) | 12 (8) | 14 (9) | 11 (8) | 15 (11) | 7 (6) | 8 (7) | 8 (7) | 11 (10) | 149 (9) | |
| No reply | 11 (6) | 17 (10) | 12 (7) | 36 (18) | 8 (5) | 9 (6) | 12 (9) | 10 (7) | 7 (6) | 9 (8) | 9 (8) | 6 (5) | 146 (8) | |

*The study started with n=29 participants, but participant F7 dropped out at Week 4 and participant F13 withdrew in Week 12. They were dropped from the final sample.

**Supplementary Table 4.** Response rates to SMART text message across all modules during the 12-week period (*n* = 27)

| Participants’ reply | Core modules | | | |  | Optional modules | |  |
| --- | --- | --- | --- | --- | --- | --- | --- | --- |
|  | Nutrition | Physical activity | Healthy stress coping | Weight management |  | Quitting smoking | Monitoring BGL | |
|  | N (%) | N (%) | N (%) | N (%) |  | N (%) | N (%) | |
| Yes | 257 (63) | 209 (53) | 183 (58) | 275 (62) |  | 34 (57) | 58 (53) | |
| No | 78 (19) | 117 (30) | 83 (25) | 90 (20) |  | 10 (17) | 45 (42) | |
| Unsure | 38 (9) | 28 (7) | 24 (8) | 43 (10) |  | 13 (21) | 3 (3) | |
| No reply | 37 (9) | 39 (10) | 28 (9) | 37 (8) |  | 3 (5) | 2 (2) | |

1. Hibbard JH, Mahoney ER, Stockard J, Tusler M. Development and testing of a short form of the patient activation measure. Health services research. 2005;40(6p1):1918-30.

2. Hibbard JH, Stockard J, Mahoney ER, Tusler M. Development of the Patient Activation Measure (PAM): conceptualizing and measuring activation in patients and consumers. Health services research. 2004;39(4p1):1005-26.

3. Aronson R, Li A, Brown RE, et al. Optimizing diabetes self-management using the novel skills, confidence, and preparedness index (SCPI). Diabetes Care. 2019;42(10):1873-8.

4. Rosenbaum S, Morell R, Abdel-Baki A, et al. Assessing physical activity in people with mental illness: 23-country reliability and validity of the simple physical activity questionnaire (SIMPAQ). BMC psychiatry. 2020;20:1-12.

5. Fernández-Ballart JD, Piñol JL, Zazpe I, et al. Relative validity of a semi-quantitative food-frequency questionnaire in an elderly Mediterranean population of Spain. British journal of nutrition. 2010;103(12):1808-16.

6. Martínez-González MA, García-Arellano A, Toledo E, et al. A 14-item Mediterranean diet assessment tool and obesity indexes among high-risk subjects: the PREDIMED trial. 2012.

7. Buysse DJ, Reynolds III CF, Monk TH, et al. Quantification of subjective sleep quality in healthy elderly men and women using the Pittsburgh Sleep Quality Index (PSQI). Sleep. 1991;14(4):331-8.

8. Schwarzer R, Renner B. Health-specific self-efficacy scales. Freie Universität Berlin. 2009;14:2009.

9. Hancock N, Bundy A, Honey A, et al. Recovery Assessment Scale-Domains & Stages (RAS-DS). 2013.

10. Hancock N, Scanlan J, Bundy A, Honey A. Recovery Assessment Scale-Domains & Stages (RAS-DS) Manual - Version 3. Sydney: University of Sydney; 2019.

11. Gonzalez-Ramirez LP, De la Roca-Chiapas JM, Colunga-Rodriguez C, et al. Validation of health behavior and stages of change questionnaire. Breast Cancer: Targets and Therapy. 2017:199-205.

12. Busner J, Targum SD. The clinical global impressions scale: applying a research tool in clinical practice. Psychiatry (edgmont). 2007;4(7):28.

13. Osborne RH, Batterham RW, Elsworth GR, et al. The grounded psychometric development and initial validation of the Health Literacy Questionnaire (HLQ). BMC public health. 2013;13:1-17.

14. Brown TA, Chorpita BF, Korotitsch W, Barlow DH. Psychometric properties of the Depression Anxiety Stress Scales (DASS) in clinical samples. Behaviour research and therapy. 1997;35(1):79-89.

15. Lovibond PF, Lovibond SH. The structure of negative emotional states: Comparison of the Depression Anxiety Stress Scales (DASS) with the Beck Depression and Anxiety Inventories. Behaviour research and therapy. 1995;33(3):335-43.

16. Nasreddine ZS, Phillips NA, Bédirian V, et al. The Montreal Cognitive Assessment, MoCA: a brief screening tool for mild cognitive impairment. Journal of the American Geriatrics Society. 2005;53(4):695-9.

17. MoCA Cognition. MoCA cognition; 2023 [Available from: <https://mocacognition.com/>.

18. Weiner BJ, Lewis CC, Stanick C, et al. Psychometric assessment of three newly developed implementation outcome measures. Implementation science. 2017;12:1-12.
